# A mobile application improves therapy-adherence rates in elderly patients undergoing rehabilitation: A crossover design study comparing documentation via iPad with paper-based control

**DOI:** 10.1097/MD.0000000000004446

**Published:** 2016-09-09

**Authors:** Alexander Mertens, Christopher Brandl, Talya Miron-Shatz, Christopher Schlick, Till Neumann, Andreas Kribben, Sven Meister, Clarissa Jonas Diamantidis, Urs-Vito Albrecht, Peter Horn, Stefan Becker

**Affiliations:** a Institute of Industrial Engineering and Ergonomics of RWTH Aachen University, Aachen, Germany; b Center for Medicine in the Public Interest, New York City, NY; c Marketing Department, Business School, Ono Academic College, Kiryat Ono, Israel; d Department of Cardiology, University Duisburg-Essen, Essen, Germany; e Institute for Drug Safety, University Hospital Essen, Essen, Germany; f Department of Nephrology, University Duisburg-Essen, Essen; g Fraunhofer Institute for Software and Systems Engineering, Dortmund, Germany; h School of Medicine, Divisions of General Internal Medicine and Nephrology, Duke University, Durham, NC; i Peter L. Reichertz Institute for Medical Informatics, University of Braunschweig—Institute of Technology and Hannover Medical School, Hannover, Germany; j Institute for Transfusion Medicine, University Hospital Essen, Essen, Germany.

**Keywords:** drug therapy, elderly patients, mobile application, self-management, tablet computer, therapy adherence

## Abstract

Medication adherence is crucial for success in the management of patients with chronic conditions. This study analyzes whether a mobile application on a tablet aimed at supporting drug intake and vital sign parameter documentation affects adherence in elderly patients.

Patients with coronary heart disease and no prior knowledge of tablet computers were recruited. They received a personal introduction to the mobile application Medication Plan, installed on an Apple iPad. The study was conducted using a crossover design with 3 sequences: initial phase, interventional phase (28 days of using the app system), and comparative phase (28 days of using a paper diary). Users experienced the interventional and comparative phases alternately.

A total of 24 patients (12 males; mean age 73.8 years) were enrolled in the study. The mean for subjectively assessed adherence (A14-scale; 5-point Likert scale, from “never” to “very often” which results in a score from 0 to 56) before the study was 50.0 (SD = 3.44). After both interventions there was a significant increase, which was more pronounced after the interventional phase (54.0; SD = 2.01) than after the comparative phase (52.6; SD = 2.49) (for all pairs after both interventions, *P* <0.001). Neither medical conditions nor the number of drug intake (amount and frequency of drug taking) per day affected subjective adherence. Logging data showed a significantly stronger adherence for the medication app than the paper system for both blood pressure recordings (*P* <0.001) and medication intake (*P* = 0.033). The majority of participants (n = 22) stated that they would like to use the medication app in their daily lives and would not need further assistance with the app.

A mobile app for medication adherence increased objectively and subjectively measured adherence in elderly users undergoing rehabilitation. The findings have promising clinical implications: digital tools can assist chronic disease patients achieve adherence to medication and to blood pressure measurement. Although this requires initial offline training, it can reduce complications and clinical overload because of nonadherence.

## 1 Introduction

Management of cardiovascular risk factors is a key issue in the secondary prevention of ischemic heart disease (IHD), which is among the leading causes of death in the Western world.^[1]^ Cardiac rehabilitation in patients with coronary heart disease comprising risk assessment and management of comorbidities, lifestyle changes, and psychosocial support has been shown to reduce mortality.^[2–4]^ However, adherence to self-management and medication remains a challenge, particularly in the management of hypertension and in elderly patients with high comorbidity and reduced awareness of their medical condition.^[5]^ Complexities of daily life, shifting priorities, and frequent polypharmacy are likely to contribute to patients’ inability to deal adequately with their medical conditions. Indeed, with respect to important risk factors of IHD such as diabetes, hypertension, and dyslipidemia, it is known that up to 50% of patients will stop taking medication for these conditions during the first year of prescription.^[6–10]^ Novel strategies are required to better address the needs of elderly and chronically ill patients.^[11]^

Mobile information technology may offer new solutions to better meet these needs. With >1 billion users having access to mobile broadband Internet and the mobile app market growing rapidly, the stakeholders involved have high hopes that this technology may improve health care.^[12]^ Expectations range from overcoming structural barriers via access in low-income countries to more effective interactive treatment of chronic conditions. Yet, previous work suggests that even when sophisticated technology is available, older users (e.g., age ≥50 years) find their initial experiences with medication applications frustrating.^[13]^ Given the importance of adherence to medication, the technological promise, and with it, the potential difficulty of older patients in using technological solutions, a study was required that would directly compare the effectiveness of advanced technological measures for increasing adherence to medication with more traditional measures, such as a pen and paper journal of medication intake. To further investigate this issue, the Institute of Industrial Engineering and Ergonomics of RWTH Aachen University initiated a usability study of the iNephro Medication Plan app, which had been developed by the Department of Nephrology and the Institute for Drug Safety, University Hospital Essen.

This study focused on whether a mobile application to support the therapy management will be accepted by elderly patients with chronic conditions and would improve their therapy adherence. Prespecified main endpoints of the statistical analysis were the effect on participants’ reported adherence to medication and to protocol blood pressure measurements. Usage patterns were quantified both through users’ subjective assessments and objective information acquired from the logged interaction protocols. In addition, the influence on affinity for technology was evaluated.

## 2 Materials and methods

### 2.1 Ethics

The Ethics Committees of RWTH Aachen University as well as the Ethics Committee of the Medical Faculty of Essen University were consulted and ethics approval issued (EK 340/14 and 14-5842-BO, respectively).

### 2.2 Study design

**Figure 1:**
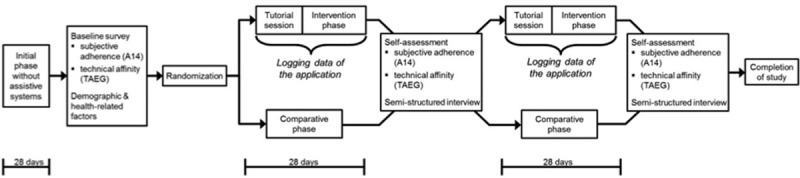
Visualization of the different phases of the study and obtained data.

The study was conducted using a crossover design with 3 sequences: an initial phase without assistive systems (between 3 and 6 months in line with standard rehabilitation treatment after an inpatient hospital stay), an interventional phase (28 days of using the app system), and a comparative phase (28 days of using a paper diary) (Fig. 1). Users experienced the interventional and comparative phases alternately: half of the users were randomly assigned to each group and switched after 28 days.

### 2.3 Recruitment

Cardiac patients were recruited via local cardiac-rehab sports groups (phase III rehabilitation) by the Institute of Industrial Engineering and Ergonomics of RWTH Aachen University. Participants were required to be at least 60 years of age with a minimum visual acuity of –0.75, which was confirmed by a mobile visual acuity screening tool with participants permitted to wear any vision aids that they use on a regular basis.^[14]^ The investigator visited the groups himself and presented the concept of the study to 100 patients. Of these, 27 patients were willing to participate, 2 of whom possessed a smartphone and 1 did not have sufficient visual acuity. Therefore, these 3 participants were excluded from the study. All patients participated voluntarily. This study was conducted independently and irrespectively of any medical treatment these patients were already receiving. No financial compensation was given for participation. Informed consent was a precondition for participation.

### 2.4 Tested system and introductory session for users

**Figure 2:**
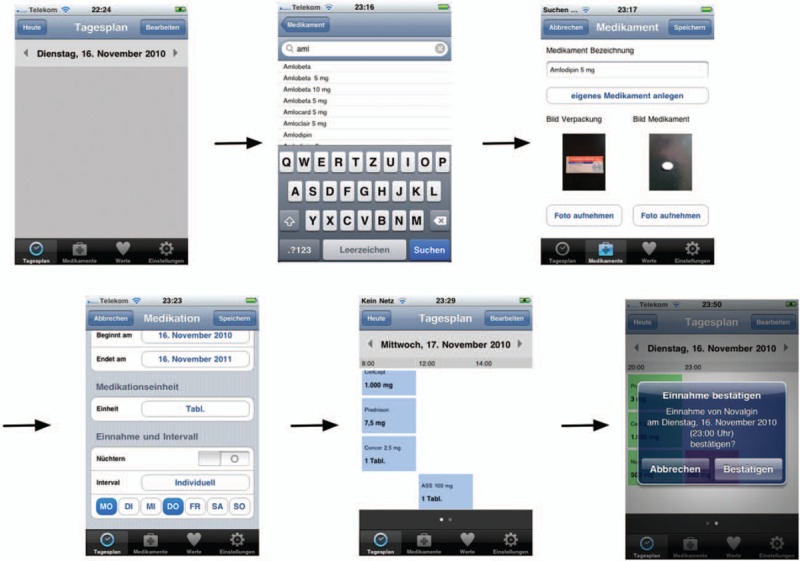
Generating a medication plan on the smartphone.^[14]^ Translation German–English: “Tagesplan” (day's schedule), “Dienstag” (Tuesday), “November” (November), “Heute” (today), “Bearbeiten” (edit), “Medikamente” (drugs), “Abbrechen” (cancel), “Speichern” (save), “Medikament Bezeichnung” (medication description), “Eigenes Medikament anlegen” (creating a custom drug), “Bild Verpackung” (picture package), Bild Medikament (picture medication), “Foto aufnehmen” (take picture), “Medikamente” (drugs), “Werte” (values), “Einstellungen” (settings), “Beginnt am” (starting at), “Endet am” (ends at), “Einheit” (unit), “Nüchtern” (sober), “Mo” (Monday), “Di” (Tuesday), “Mi” (Wednesday), “Do” (Thursday), “Fr” (Friday), “Sa” (Saturday), “So” (Sunday), “Einnahme bestätigen” (confirm intake).

This usability trial studied the Medication Plan app (version 1.3) on a first-generation Apple iPad (iOS version 5.1.1).^[15,16]^ The app specifications, such as being able to set reminders for a number of medications, supported the drug intake needs of patients with chronic conditions on polypharmacy (see video tutorial at http://www.youtube.com/watch?v=nui78JqwMHE and Fig. 2). The home screen of the test iPad was modified so that only the Medication Plan app was available in the dock, and all further standard applications were placed in a folder on the second menu page. The possibility to delete applications in the device's restrictions settings was disabled. As a further small modification, we put a green sticker on the iPad's home button to help users locate it easily.

### 2.5 Study implementation

Users were visited at home 3 times by the same investigator to be introduced to each system (the initial phase, the iPad intervention, and the comparative pen and paper phase) and to complete the questionnaires. In addition, semi-structured interviews were performed during each visit to assess participants’ responses to using the system. Each of the data collection sessions took between 30 minutes and 2 hours. All of the materials were in German, the native language of the participants. After the participants completed the initial questionnaires, the examiner listed medications presently prescribed on a prepared form, based on the patients’ self-report. The following data were recorded: name of the medication, number of intakes per day, and corresponding doses. Before the intervention phase, the examiner entered participants’ medication into the app “Medication Plan.” The participants were introduced to the iPad and iNephro application in an interactive learning-by-doing tutorial session. As none of the patients had used a tablet computer or smartphone before, we also explained the general concepts of tapping and swiping. The participants were familiarized with the functions of the application (i.e., confirming medication intake and recording blood pressure values) and how to recover if a wrong input had been made. The volume of the acoustic pill reminder was set as desired by the users in the test groups: they could choose how loud or quiet it would be. They were also instructed how to adjust it later on by themselves. The users also received a paper-based notepad in which they could write down any problems or particularly positive aspects (e.g., those which might have been difficult at first) they encountered when utilizing the system. The intervention was not used in the context of a particular medical treatment, and no feedback by a doctor was given on recorded vital signs. Throughout the comparative phase, participants used a self-explanatory paper diary as a control method to log their medication intake and blood pressure values.

### 2.6 Objective indicators for technology adherence

Confirmation rates of medication intake and the number of blood pressure records in the iPad intervention and in the paper diary were analyzed. In order to be able to compare the outcomes of the interventions, we did a target–performance comparison. For medication, the target was defined as the number of medications each participant had to take each day multiplied by the days they actually used the system or the diary. The variable “absolute performance” was defined as the actual number of confirmations by the patient that they took the medication. Having to take >1 unit (i.e., >1 pill) of a single medication at the same point in time or on the same day was considered as 1 medication intake (“all or none”) as the application cannot register whether participants only took a subset of a particular medication. The rate of adherence to confirm medication then is the ratio of performance and target multiplied by 100 to obtain a percentage value. Assessing adherence for blood pressure recording was different as participants could take more blood pressure records each day than actually agreed upon. For instance, if a user had to take 1 record each day and actually took 2 records on 1 day and none the next, he would still score an adherence rate of 100%. To exclude this factor, the adherence was estimated for each day, capped at 100%, and then averaged.

### 2.7 Questionnaires

Technical knowledge and experience were assessed using an adapted version of the computer literacy scale (CLS). The score on the scale allowed us to categorize the participants as novices, intermediates, and experts accordingly.^[17]^

Subjective adherence was determined by the A14-scale, which contains 14 items that ask participants to report on a 5-point Likert scale, ranging from “never” (0) to “very often” (4), their behavior with respect to medication adherence and the degree to which various barriers to adherence apply to them.^[18]^ Based on the A14-scale, values <50 are regarded as nonadherent and values between 50 and 56 as adherent (sums ranging between 0 and 56).

Affinity for technology was assessed using the TA-EG questionnaire which is designed to assess a person's positive attitude, excitement, and trust toward technology.^[19]^ The TA-EG consists of 19 statements on different aspects of technology rated on a 5-point Likert scale, ranging from “do not agree at all” to “completely agree.”

Semi-structured interviews were constructed around central questions, including how participants incorporated the iPad-delivered intervention in their daily lives and what they liked or disliked about it.^[20]^ Questions were adapted to norm based upon ISO 9241.

### 2.8 Data collection and analysis

Data was analyzed using SPSS statistics software version SPSS 21.0 (IBM, Armonk, New York). A multifactorial analysis of variance (ANOVA) with repetitions for the different factor levels of the response variables with a significance level of 0.05 was conducted. Significant findings were additionally analyzed by post hoc analysis and Bonferroni correction to minimize the Type-I error rate because of multiple paired comparisons of mean values. Sphericity was assessed by Mauchly's test. In violation of Mauchly's test, the corrected value according to Greenhouse–Geisser was used. For significant effects, the effect size ω^2^ was determined for the main effects of the repeated measurements using the following equation:^[21]^ 
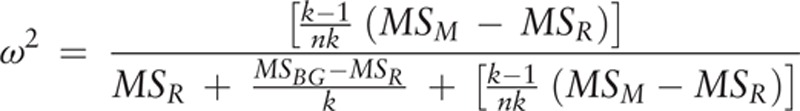


Here the variables *MS*_*M*_ and *MS*_*R*_ stand for “mean square for the model” or “residual mean square” and describe the mean square for the experimental factors, respectively, the error of each factor. The effect sizes can take values between 0 and 1, where ω^2^ = 0.01 describes a small effect, ω^2^ = 0.06 a medium effect, and ω^2^ = 0.14 a strong effect. ^[21–23]^

## 3 Results

A total of 24 users were enrolled in the study (12 males) with a mean age of 73.8 years (SD = 7.5). All participants suffered from a coronary heart disease or had experienced myocardial infarction requiring inpatient hospital stay within 6 months before the study. On average 2.2 (SD * = * 0.9) additional chronic conditions were reported per patient: hypertension (n = 14), dyslipidemia (n = 9), diabetes (n = 9), liver (n = 2), and lung disease (n = 2). The 24 participants within the cohort had been instructed by their physician to take drugs between 2 and 6 times a day with an average of 3.8 drugs (SD = 1.4). Furthermore, as part of their care, all the participants were asked by their consulting physician to take blood pressure readings between 1 and 4 times a day (*M* = 2.0; SD = 0.9) (Table 1). All participants were retired and lived independently at home. None of them were in need of assistance with daily activities (Table 1). None had prior experience with a smartphone or tablet, but 14 owned a computer and 17 used the Internet regularly. The within-subject contrasts of sex and age were balanced within the cohort and used as a control variable.

**Table 1:**
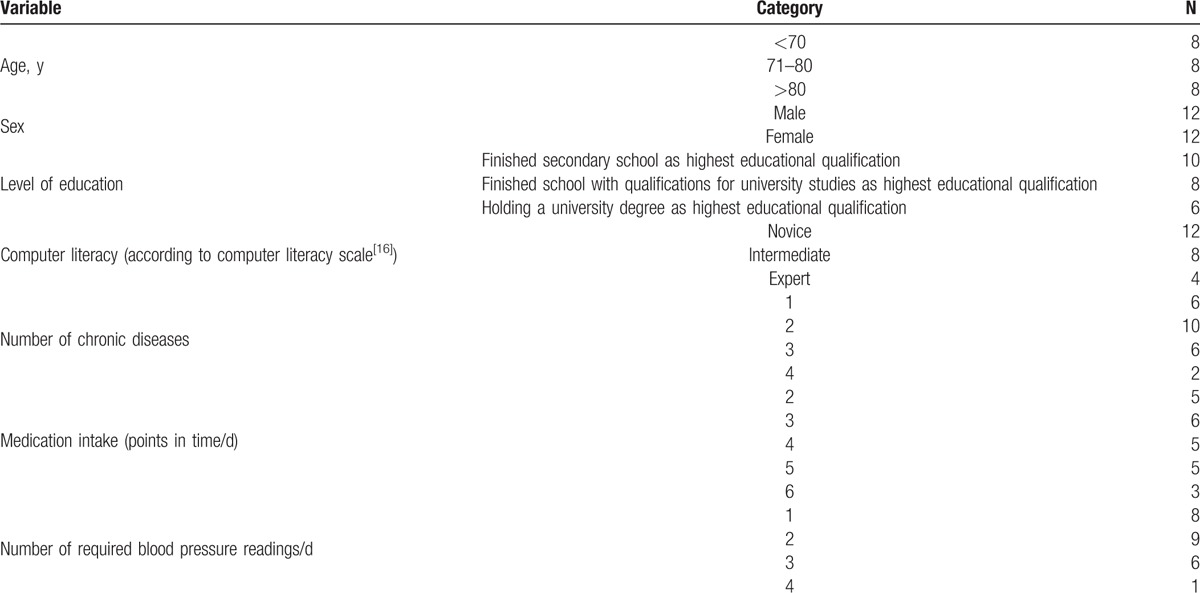
Characteristics of the cohort.

### 3.1 Subjective adherence

**Figure 3:**
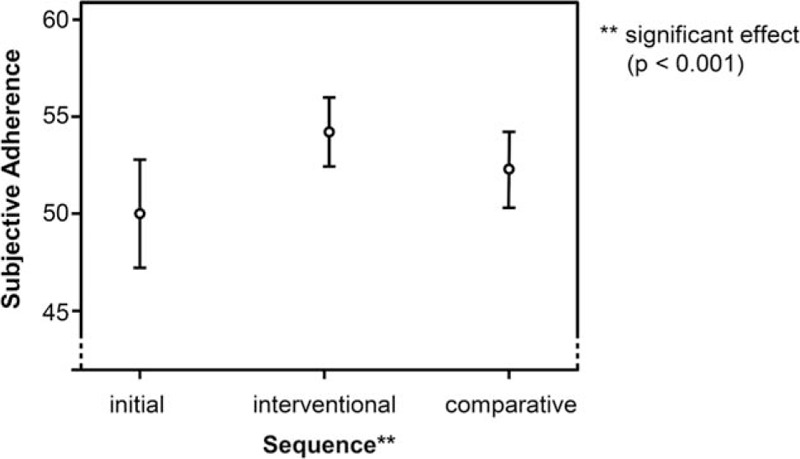
Subjective adherence of the participants after initial, interventional, and comparative phase. Range for subjective adherence from 0 to 56; values <50 are regarded as nonadherent.

Mean subjectively assessed adherence before the study without a supporting system was 50.02 (SD = 3.44), after the interventional phase (medication app) 53.96 (SD * = * 2.01), and after the comparative phase (paper diary) 52.60 (SD = 2.49). Inferential analysis of the 3 measurement points confirmed a significant effect of the respective type of intervention (*F* = 31.662; *df* = 1.613; *P* <0.001; Fig. 3) with a medium effect size of ω^2^ = 0.07. Post hoc pairwise analysis with Bonferroni correction showed significant differences between both interventions (*P* = 0.02) and in comparison to the initial phase (both *P* <0.001). The effect on adherence was more pronounced after the medication app intervention than after the paper diary. The following variables: individual medical conditions and therapy, represented by the quantity of chronical diseases (*F* = 2.494; *df* = 3; *P* = 0.106), the number of drug intakes per day (*F* = 0.994; *df* = 4; *P* = 0.627), and the average daily number of the required vital parameter readings (*F* = 1.583; *df* = 3; *P* = 0.515), were not associated with the improved subjective evaluation of adherence to medication.

### 3.2 Objective adherence

**Figure 4:**
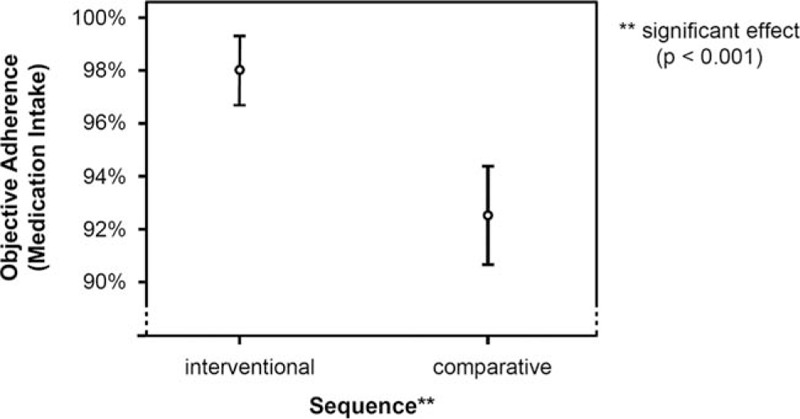
Analysis of documentation of medication intake.

**Figure 5:**
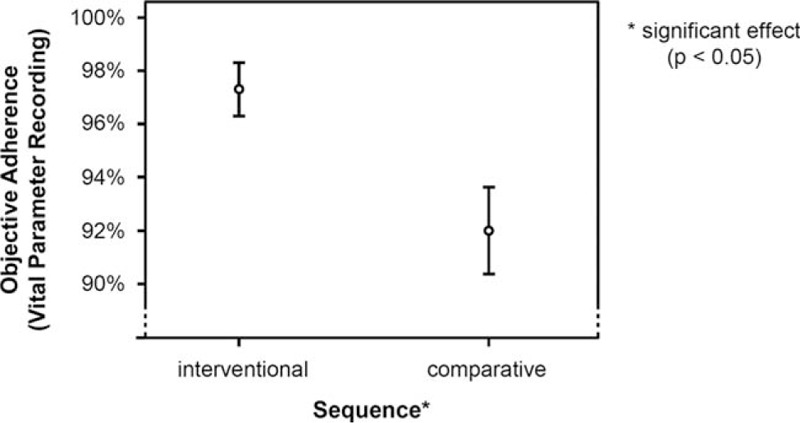
Analysis of vital parameter (blood pressure) recordings.

Analysis of the logging data of the iPad application and the documented medication intake of the paper journal with regard to medication intake showed a significantly stronger adherence for the app system than the paper diary system (*F* = 27.404; *df* = 1; *P* <0.001), with a medium effect size of ω^2^ = 0.09 (Fig. 4). Medication intake/day (*F* = 0.072; *df* = 4; *P* = 0.980), the number of chronic diseases (*F* = 2.521; *df* = 3; *P* = 0.244), as well as required blood pressure readings (*F* = 0.641; *df* = 3; *P* = 0.700), did not affect the “recording” adherence for medication intake. Documentation of vital parameter (blood pressure) recordings showed significantly stronger adherence while using the app, relative to the paper system (*F* = 361.349; *df * =  1; *P* = 0.033) although the effect size was small, ω^2^ = 0.05 (Fig. 5). Similar to the influence on the medication intake, the documentation of vital parameter (blood pressure) recordings was not affected by the number of chronic diseases (*F* = 1.882; *df* = 2; *P* = 0.458), medication intake/day (*F* = 11.748; *df* = 4; *P* = 0.215), and the number of required blood pressure readings (*F* = 3.138; *df* = 3; *P* = 0.388).

### 3.3 Technical affinity

Technical affinity differed significantly after each intervention (*F* = 13.538; *df* = 2; *P* = 0.003). The correlation between the type of assistance (tablet or paper diary) and the technical affinity had a medium effect size of ω^2^ = 0.07. Further, paired testing showed significant differences between all values (naive vs tablet experienced: *P* <0.001; naive vs paper diary experienced: *P* = 0.043; tablet experienced vs. paper diary experienced: *P* = 0.002). As expected, the 28-day interventional phase with assistance through the tablet computer had the strongest impact on technical affinity.

### 3.4 Impact of comorbidities on usage

All of the participants were required by their physicians to measure their blood pressure at least once a day. Users suffering from hypertension were significantly more adherent to the functionality of vital sign documentation than other participants (*F* = 480.720; *df* = 1; *P* = 0.036, small effect size ω^2^ = 0.05), whereas there was no significant effect on confirmation of medication intake compared with users without this condition (*F* = 35.98; *df* = 1; *P* = 0.131). Other conditions like diabetes and dyslipidemia did not affect technical adherence.

### 3.5 Interviews on user experience

The vast majority of participants (n = 22) stated in structured interviews that they would like to use the medication app in everyday life and would not need further assistance with using it. One of the participants had a red–green color deficiency but had no problems telling apart the different colors of the status of the medication intake (i.e., red, green, and blue). Four mentioned they had accidentally deleted one of their blood pressure records. All users needed between 1 and 6 minutes per day to use the system. Only 3 of them required the manual provided for additional help. More than half (n = 13) used the diagram function of the blood pressure values: 4 used it from time to time and 6 did not use the diagram function at all. Three said they did not use the function because they were not able to interpret them: “I took a look but I don’t know what to do with it. Without my doctor, I’m not able to explain this graph.” The participants who did use the diagram function said that “it is useful to spot peaks,” “it is easy to see whether it is a random outlier or a trend,” and “it is much clearer than looking at the numbers.” Further aspects regarding acceptance, ease/joy of use, and user interaction are summarized in Table 2.

**Table 2:**
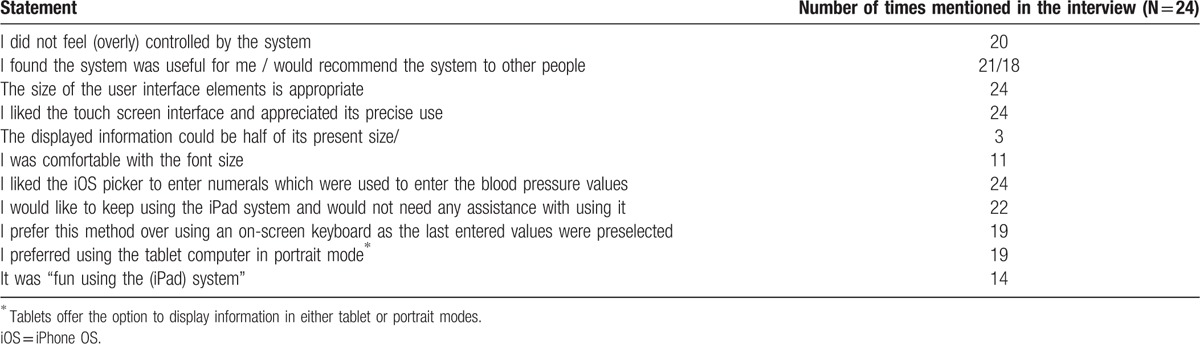
User feedback regarding acceptance, ease/joy of use and user interaction with the mobile application “Medication Plan.”.

## 4 Discussion

The present study is the first to compare a medication app and paper diary intervention to support therapy adherence among cardiac patients. The present study suggests that such an intervention may be of benefit to improve therapy adherence, although the exact degree to which the findings hold in broader populations needs to be examined. Findings from previous research on the efficacy of mobile phone reminders and electronic home devices to improve therapy adherence have been mixed, suggesting that the use of these technologies alone is insufficient to significantly improve patient outcomes.^[24–30]^ When automated reminders using mobile phones or electronic home devices are used in combination with in-person reminders from medical care providers, the results have been more encouraging.^[24]^ Even so, such interventions have been described as “plagued by high resource intensity, lack of specificity regarding content and delivery, and impracticality for everyday clinical practice settings.”^[24]^ By contrast, our intervention has been scalable and proved itself to be highly practical in the context of chronic patients’ homes, albeit ones with high initial adherence levels. Moreover, there is a paucity of research studies that have directly compared method of adherence with one another, and the present study attempts to close this gap.^[31]^

We demonstrate how, in a self-selected group of patients who volunteered to participate in the study and were quite adherent to medication in the first place, the interventions improved adherence. “Adherence” in our study carried several meanings—a subjective sense of adherence to medication, as well as actual adherence in using the medication app intervention, and reported adherence of performing blood pressure measurements. The interventions improved all the related types of adherence, though the iPad-delivered intervention resulted with greater adherence than the pen and paper journal. Digital interventions and reminder systems carry an enormous potential for scalable ways of reaching a wide range of patient populations, as well as healthy populations that need support in maintaining recommended nutrition and exercise regimens.

Such work, of introducing a digital intervention, may raise 3 difficult questions, all of which were successfully answered by the present work. The first question is—would older patients find an iPad intervention convenient to use or maybe a more traditional pen and paper intervention would be better received? Our results unequivocally suggest that the participants’ adherence improved more with the digital intervention than with the pen and paper one. Digital intervention via text messaging has been used to help aid tobacco cessation, improve physical activity and stimulate weight management efforts, and improve diabetic control, among countless other applications.^[25,26]^ Further, studies from various clinical contexts in which text messaging services were introduced reported improved medication adherence.^[27–30]^ However, the digital and traditional intervention comparison as presented in our study is unique and can help guide clinics and public health officials in designing future interventions.

The second question is—would this type of digital intervention only appeal to and be beneficial for more technologically savvy patients? None of the patients who chose to participate had previously used a tablet or a smartphone, and yet, following a short instruction session, they were able to use the iPad intervention independently. Interestingly, technical affinity of the participants increased following the iPad intervention. This might be another benefit of the study, namely, helping to remove barriers between older patients and new technology. Such a goal would be especially important to achieve given that the study was conducted in Germany, whereas senior usage of mobile technology in the 60-plus segment in neighboring countries is considerably different. For example, in Switzerland, more than half of the population aged between 55 and 69 years uses mobile communication technology.^[32]^ Even when disregarding age, Germany lags in general usage of smartphones behind Spain, Italy, Canada, the United States, and the United Kingdom.^[33]^ This lack of knowledge may reduce the intention to use apps in everyday life. Previous studies confirmed that the below average utilization of such applications is caused by a lack of acceptance within the target group.^[34,35]^ On the contrary, we were able to demonstrate that after a relatively short introductory session users could handle a mobile device with relative ease and did not need further assistance. Our findings give additional insights into what has been termed the “digital divide”,^[36]^ a term coined by developers, which implies that compared with the younger generation, older individuals are less likely to make extensive use of digital technology.^[16]^ The insights are that the digital divide can be mitigated by providing offline training on digital tools. Indeed, once this is done with older participants, they are likely to persist with the digital tools over time. We caveat this by mentioning again that our participants were a self-selected group, which may have been more motivated, and less technology averse than other older patients with chronic diseases.

The third question is—can a medication app improve adherence to medication over time? We have demonstrated that it can, over a period of 28 days. Interviews with the participants provide strong support in favor of a medication app, as an overwhelming majority of our participants indicated their willingness to continue using the app once the study period was over. Direct assessment of user activity over time can offer objective and more accurate data on the usefulness of a mobile application.

### 4.1 Limitations

Several limitations exist in the present inquiry. In our study, medication intake needed to be confirmed via iPad. Even though this was a simple procedure, reports do not necessarily guarantee medication intake, and vice versa. A patient may report taking the medication, but not actually take it. Conversely, a patient may have forgotten to report an intake. In spite of this limitation, the basic principle of our intervention method is very similar to pill counting, where patients document their behavior and there is no external “quality control” for the entries. Clinical studies in pill counting have shown that the patient-reported data give an insight into the level of adherence.^[37]^ That is why we assume there is similar reliability and validity for our iPad intervention.

Another limitation is the length of the study. It extended over a period of 56 days per user, which is relatively short considering the fact that we examined people who need to take medication for the rest of their lives. Thus, although we show the effectiveness of the intervention over a 56-day period, we cannot ascertain that the intervention will continue to be effective in the long run. However, other mobile phone research suggests that time has an effect on how users evaluate a new device and its applications. When examining evaluations given over a period of 5 months, researchers find that, over time, users place less importance on user experience and enjoyment and greater importance on usability.^[38]^ This leads us to believe that over time patients would continue to use the app because of the high usability value.

With regard to objective adherence in our study, it was found to be very high. Remarkably, the iPad intervention improved medication intake over the already high initial therapy adherence as well as the positive effect on medication intake achieved with the paper diary. It is perhaps even more remarkable considering that a review has found less than half the adherence interventions to be effective and found that the interventions that were effective over time were complex, involving reminders, counseling, and more, whereas our intervention was only based on one means of aiding patients.^[39]^ In the next phase of research medication adherence should be monitored using electronic monitoring methods such as monitored pill bottles (e.g., Medication Event Monitoring System (MEMS) cap), so that the correspondence of objectively monitored medication adherence with self-reported medication taking behavior using the app could be verified. This would also allow for a baseline with no intervention. Obviously, these suggestions are for the future and beyond the scope of the study.

Adherence is a multidimensional problem and cannot solely be solved by Apple's promise “there is an app for that,” which is how it introduced its App Store in 2008. In this work, we demonstrated that a mobile application for medication adherence increased objectively and subjectively measured adherence compared with baseline and with a paper diary intervention in elderly users undergoing phase III cardiac rehabilitation. Patients, whose average age was >70 years, indicated a willingness to continue using the app. Although the app was supplemented with a face-to-face initial training stage, no additional technical support was required. The clinical implication is that mobile technology, combined with offline support, can be an effective tool for promoting adherence to medication with elderly populations. It is furthermore promising that users’ lack of technological savviness can be overcome by a training phase. This suggests that if patients receive adequate technological support, digital tools can be an integral part of their therapy management. Such tools can then be used over time in rehabilitation and can also benefit care of patients with chronic conditions in general.
